# Cinnarizinium fumarate

**DOI:** 10.1107/S1600536812051239

**Published:** 2012-12-22

**Authors:** C. N. Kavitha, Sema Ōztūrk Yildirim, Jerry P. Jasinski, H. S. Yathirajan, Ray J. Butcher

**Affiliations:** a Department of Studies in Chemistry, University of Mysore, Manasagangotri, Mysore 570 006, India; b Department of Physics, Faculty of Sciences, Erciyes University, 38039 Kayseri, Turkey; c Department of Chemistry, Keene State College, 229 Main Street, Keene, NH 03435-2001, USA; d Department of Chemistry, Howard University, 525 College Street NW, Washington, DC 20059, USA

## Abstract

In the title salt {systematic name: 4-diphenyl­methyl-1-[(*E*)-3-phenyl­prop-2-en-1-yl]piperazin-1-ium (2*Z*)-3-carb­oxy­prop-2-enoate}, C_26_H_29_N_2_^+^·C_4_H_3_O_4_^−^, the piperazine ring in the cation adopts a distorted chair conformation and contains a positively charged N atom with quaternary character. The dihedral angle between the mean planes of the phenyl rings of the diphenyl­methyl group is 74.2 (7)° and those between these rings and the phenyl ring of the 3-phenyl­prop-2-en-1-yl group are 12.7 (9) and 80.6 (8)°. In the crystal, N—H⋯O and O—H⋯O hydrogen bonds form chains along [001]. Weak C—H⋯O inter­actions connect parallel chains along [010], forming layers perpendicular to the *a*-axis direction.

## Related literature

For cinnarizine as a calcium channel blocker, see: Terland & Flatmark (1999 ▶), as a nootropic drug, see: Towse (1980 ▶) and for a clinical evaluation in various allergic disorders, see: Barrett & Zolov (1960 ▶). For related structures, see: Bertolasi *et al.* (1980 ▶); Dayananda *et al.* (2012 ▶); Jasinski *et al.* (2011 ▶); Mouillé *et al.* (1975 ▶); Siddegowda *et al.* (2011 ▶); Song *et al.* (2012 ▶). For puckering parameters, see: Cremer & Pople (1975 ▶). For standard bond lengths, see: Allen *et al.* (1987 ▶).
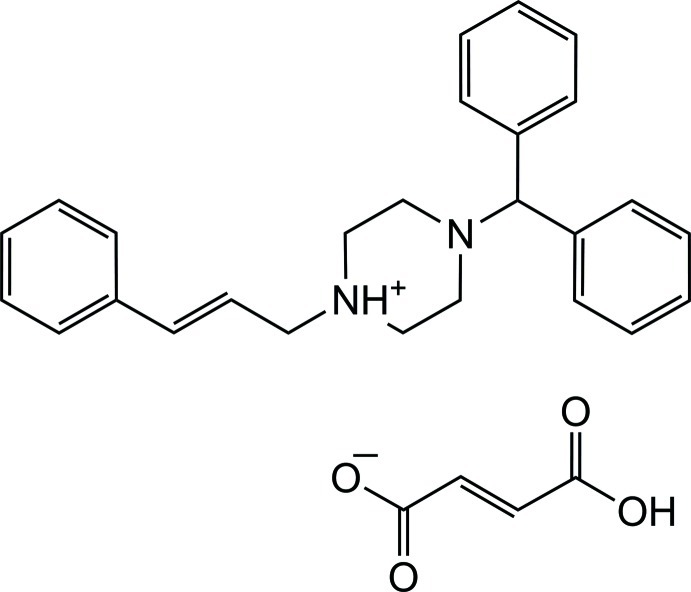


## Experimental

### 

#### Crystal data

C_26_H_29_N_2_^+^·C_4_H_3_O_4_^−^
*M* *_r_* = 484.58
Monoclinic, 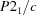

*a* = 21.9467 (4) Å
*b* = 10.43729 (18) Å
*c* = 11.20623 (19) Å
β = 90.0458 (15)°
*V* = 2566.95 (8) Å^3^
*Z* = 4
Cu *K*α radiation
μ = 0.67 mm^−1^
*T* = 123 K
0.60 × 0.30 × 0.25 mm

#### Data collection

Agilent Xcalibur (Ruby, Gemini) diffractometer
Absorption correction: multi-scan (*CrysAlis RED* and *CrysAlis PRO*; Agilent, 2011 ▶) *T*_min_ = 0.732, *T*_max_ = 1.000
9777 measured reflections
5146 independent reflections
4289 reflections with *I* > 2σ(*I*)
*R*_int_ = 0.028

#### Refinement

*R*[*F* ^2^ > 2σ(*F* ^2^)] = 0.043
*wR*(*F* ^2^) = 0.121
*S* = 1.02
5146 reflections
333 parameters
H atoms treated by a mixture of independent and constrained refinement
Δρ_max_ = 0.27 e Å^−3^
Δρ_min_ = −0.22 e Å^−3^

### 

Data collection: *CrysAlis PRO* (Agilent, 2011 ▶); cell refinement: *CrysAlis PRO*; data reduction: *CrysAlis PRO*; program(s) used to solve structure: *SHELXS97* (Sheldrick, 2008 ▶); program(s) used to refine structure: *SHELXL97* (Sheldrick, 2008 ▶); molecular graphics: *SHELXTL* (Sheldrick, 2008 ▶); software used to prepare material for publication: *SHELXTL*.

## Supplementary Material

Crystal structure: contains datablock(s) global, I. DOI: 10.1107/S1600536812051239/zl2527sup1.cif

Structure factors: contains datablock(s) I. DOI: 10.1107/S1600536812051239/zl2527Isup2.hkl

Supplementary material file. DOI: 10.1107/S1600536812051239/zl2527Isup3.cml

Additional supplementary materials:  crystallographic information; 3D view; checkCIF report

## Figures and Tables

**Table 1:** Hydrogen-bond geometry (Å, °)

*D*—H⋯*A*	*D*—H	H⋯*A*	*D*⋯*A*	*D*—H⋯*A*
N2—H1*N*⋯O4	0.943 (19)	1.740 (19)	2.6750 (15)	170.9 (17)
O1—H1*O*1⋯O4^i^	0.94 (3)	1.70 (3)	2.6270 (15)	168 (2)
C18—H18*B*⋯O3^ii^	0.97	2.56	3.3839 (18)	143
C15—H15*A*⋯O3^ii^	0.97	2.47	3.3463 (18)	151
C15—H15*B*⋯O2^iii^	0.97	2.46	3.1941 (19)	132

Symmetry codes: (i) 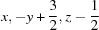; (ii) 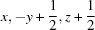; (iii) 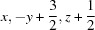.

**Fig. 1.**
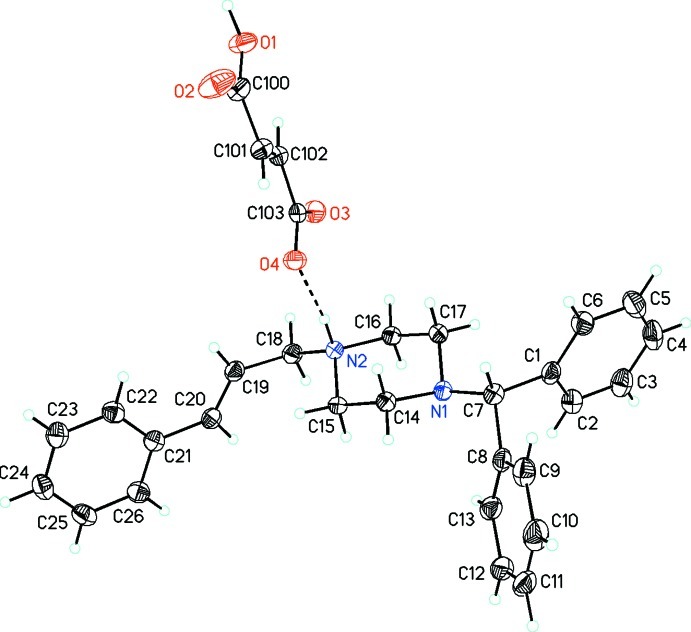
Molecular structure of the title compound showing the atom labeling scheme and 30% probability displacement ellipsoids. Dashed lines indicate N2—H2···O4 cation-anion hydrogen bonds.

**Fig. 2.**
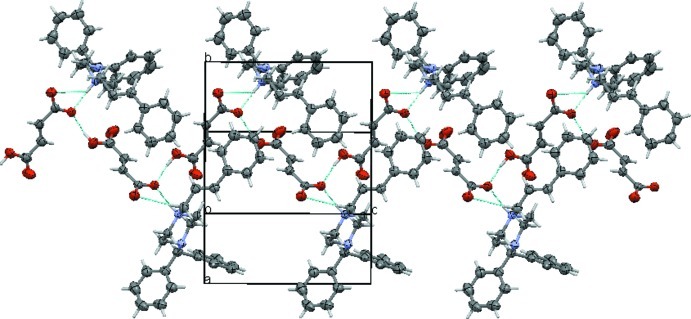
Packing diagram of the title compound viewed along the a axis. Dashed lines indicate N—H···O and O—H···O hydrogen bonds forming infinite one-dimensional chains along [001].

**Fig. 3.**
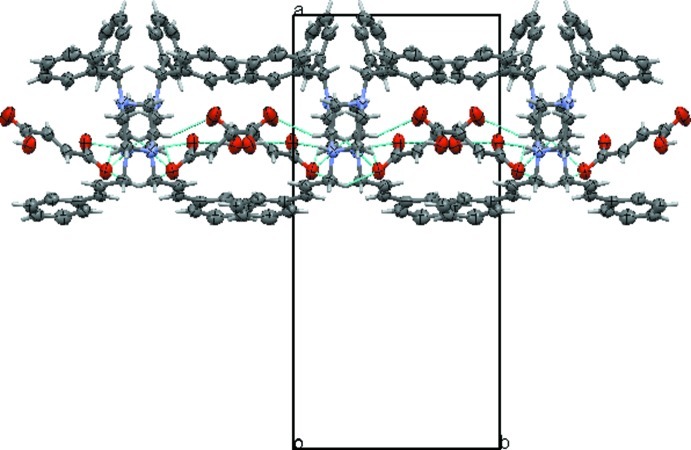
Packing diagram of the title compound viewed along the c axis. Dashed lines indicate weak C—H···O intermolecular interactions (Table 1) which are also observed connecting parallel chains along [010] to form layers perpendicular to the a-axis direction of the structure.

## supplementary crystallographic information

### Comment

Cinnarizine (1-benzhydryl-4-cinnamyl-piperazine) is a drug derivative of piperazine and a calcium channel blocker (Terland & Flatmark, 1999). Cinnarizine is an antihistamine which is mainly used for the control of nausea and vomiting due to motion sickness. It could be also viewed as a nootropic drug because of its vasorelaxating abilities (due to calcium channel blockage), which happen mostly in the brain and it is also used as a labyrinthine sedative (Towse, 1980). A clinical evaluation of cinnarizine in various allergic disorders is published (Barrett & Zolov, 1960). Cinnarizine can be used in scuba divers without an increased risk of central nervous system oxygen toxicity. The crystal structures of some related compounds viz., cinnarizine (Mouillé *et al.*, 1975), cyclizine hydrochloride (Bertolasi *et al.*, 1980), cinnarizinium dipicrate (Jasinski *et al.*, 2011), cinnarizinium picrate (Song *et al.*, 2012), opipramolium fumarate (Siddegowda *et al.*, 2011) and cinnarizinium 3,5-dinitrosalicylate (Dayananda *et al.*, 2012) have been reported. In continuation of our work on the salts of pharmaceutical compounds and in view of the importance of cinnarizine, this paper reports the crystal structure of the title salt, C_26_H_29_N_2_^+^ . C_4_H_3_O_4_^-^, (I).

The asymmetric unit of (I) consists of a cinnarizinium-hydrogen fumarate cation-anion pair (Fig. 1). The six-membered piperazine ring (N1/C14/C15/N2/C16/C17) in the cation adopts a distorted chair conformation with puckering parameters Q = 0.6021 (14)Å, θ = 174.02 (12)°, φ = 184.5 (13)°, (Cremer & Pople (1975)) and contains a positively charged N atom (N2) with quaternary character. The dihderal angle between the mean planes of the two diphenyl rings (C1–C6 and C8–C13)is 74.2 (7)° and that between these rings and the extended phenyl ring (C21–C26) is 12.7 (9)° and 80.6 (8)°, respectively. Bond lengths are in normal ranges (Allen *et al.*, 1987). Crystal packing is stabilized by N—H···O and O—H···O hydrogen bonds forming infinite one-dimensional chains along [001] (Fig. 2). Weak C—H···O intermolecular interactions (Table 1) are also observed connecting parallel chains along [010] (Fig. 3) to form layers perpendicular to the a-axis direction of the structure.

### Experimental

Cinnarizine (3.68 g, 0.01 mol) and fumaric acid (1.16 g, 0.01 mol) were dissolved in hot dimethyl sulphoxide solution and stirred over a heating magnetic stirrer for a few minutes. The resulting solution was allowed to cool slowly at room temperature. X-ray quality crystals of the title compound appeared after a few days. (m.p.: 468–471 K).

### Refinement

H1N and H1O1 were located by Fourier maps and refined isotropically. All of the remaining H atoms were placed in their calculated positions and then refined using the riding model with Atom—H lengths of 0.93Å (CH) or 0.97Å (CH_2_). Isotropic displacement parameters for these atoms were set to 1.19-1.21 (CH, CH_2_) times *U*_eq_ of the parent atom.

### Crystal data

**Table:**

C_26_H_29_N_2_^+^·C_4_H_3_O_4_^−^	*F*(000) = 1032
*M**_r_* = 484.58	*D*_x_ = 1.254 Mg m^−^^3^
Monoclinic, *P*2_1_/*c*	Cu *K*α radiation, λ = 1.54184 Å
Hall symbol: -P 2ybc	Cell parameters from 3517 reflections
*a* = 21.9467 (4) Å	θ = 3.9–75.4°
*b* = 10.43729 (18) Å	µ = 0.67 mm^−^^1^
*c* = 11.20623 (19) Å	*T* = 123 K
β = 90.0458 (15)°	Prism, colorless
*V* = 2566.95 (8) Å^3^	0.60 × 0.30 × 0.25 mm
*Z* = 4	

### Data collection

**Table:**

Agilent Xcalibur (Ruby, Gemini) diffractometer	5146 independent reflections
Radiation source: Enhance (Cu) X-ray Source	4289 reflections with *I* > 2σ(*I*)
Graphite monochromator	*R*_int_ = 0.028
Detector resolution: 10.5081 pixels mm^-1^	θ_max_ = 75.6°, θ_min_ = 4.0°
ω scans	*h* = −23→27
Absorption correction: multi-scan (*CrysAlis RED* and *CrysAlis PRO*; Agilent, 2011)	*k* = −12→8
*T*_min_ = 0.732, *T*_max_ = 1.000	*l* = −13→13
9777 measured reflections	

### Refinement

**Table:**

Refinement on *F*^2^	Primary atom site location: structure-invariant direct methods
Least-squares matrix: full	Secondary atom site location: difference Fourier map
*R*[*F*^2^ > 2σ(*F*^2^)] = 0.043	Hydrogen site location: inferred from neighbouring sites
*wR*(*F*^2^) = 0.121	H atoms treated by a mixture of independent and constrained refinement
*S* = 1.02	*w* = 1/[σ^2^(*F*_o_^2^) + (0.0628*P*)^2^ + 0.4518*P*] where *P* = (*F*_o_^2^ + 2*F*_c_^2^)/3
5146 reflections	(Δ/σ)_max_ = 0.001
333 parameters	Δρ_max_ = 0.27 e Å^−^^3^
0 restraints	Δρ_min_ = −0.22 e Å^−^^3^

### Special details

**Table:**

Geometry. All esds (except the esd in the dihedral angle between two l.s. planes) are estimated using the full covariance matrix. The cell esds are taken into account individually in the estimation of esds in distances, angles and torsion angles; correlations between esds in cell parameters are only used when they are defined by crystal symmetry. An approximate (isotropic) treatment of cell esds is used for estimating esds involving l.s. planes.
Refinement. Refinement of F^2^ against ALL reflections. The weighted R-factor wR and goodness of fit S are based on F^2^, conventional R-factors R are based on F, with F set to zero for negative F^2^. The threshold expression of F^2^ > 2sigma(F^2^) is used only for calculating R-factors(gt) etc. and is not relevant to the choice of reflections for refinement. R-factors based on F^2^ are statistically about twice as large as those based on F, and R- factors based on ALL data will be even larger.

### Fractional atomic coordinates and isotropic or equivalent isotropic displacement parameters (Å^2^)

**Table:**

	*x*	*y*	*z*	*U*_iso_*/*U*_eq_	
O1	0.70117 (5)	0.78316 (11)	0.33222 (10)	0.0323 (3)	
O2	0.76739 (6)	0.88857 (12)	0.44702 (12)	0.0446 (3)	
O3	0.64077 (5)	0.41055 (10)	0.57965 (9)	0.0298 (2)	
O4	0.70688 (5)	0.50977 (10)	0.69945 (9)	0.0272 (2)	
N1	0.79869 (5)	0.16984 (11)	0.86255 (10)	0.0208 (2)	
N2	0.68573 (5)	0.31174 (11)	0.84519 (10)	0.0200 (2)	
C1	0.87241 (6)	0.02312 (14)	0.76576 (13)	0.0237 (3)	
C2	0.85173 (7)	−0.09616 (15)	0.80480 (14)	0.0287 (3)	
H2A	0.8321	−0.1028	0.8780	0.034*	
C3	0.86015 (7)	−0.20467 (16)	0.73566 (16)	0.0340 (3)	
H3A	0.8461	−0.2837	0.7625	0.041*	
C4	0.88951 (7)	−0.19554 (17)	0.62621 (16)	0.0362 (4)	
H4A	0.8954	−0.2685	0.5800	0.043*	
C5	0.90995 (7)	−0.07792 (18)	0.58618 (15)	0.0367 (4)	
H5A	0.9293	−0.0716	0.5126	0.044*	
C6	0.90159 (7)	0.03164 (16)	0.65610 (13)	0.0288 (3)	
H6A	0.9156	0.1105	0.6291	0.035*	
C7	0.86398 (6)	0.14289 (13)	0.84173 (12)	0.0223 (3)	
H7A	0.8818	0.2158	0.7990	0.027*	
C8	0.89718 (6)	0.12793 (13)	0.96069 (13)	0.0241 (3)	
C9	0.95738 (7)	0.16951 (15)	0.96988 (15)	0.0315 (3)	
H9A	0.9759	0.2094	0.9052	0.038*	
C10	0.98986 (8)	0.15168 (18)	1.07497 (17)	0.0396 (4)	
H10A	1.0300	0.1796	1.0803	0.048*	
C11	0.96277 (8)	0.09271 (17)	1.17150 (16)	0.0402 (4)	
H11A	0.9847	0.0802	1.2416	0.048*	
C12	0.90255 (8)	0.05206 (15)	1.16380 (14)	0.0348 (4)	
H12A	0.8842	0.0127	1.2290	0.042*	
C13	0.86965 (7)	0.07003 (14)	1.05883 (14)	0.0280 (3)	
H13A	0.8293	0.0434	1.0542	0.034*	
C14	0.79157 (6)	0.29648 (13)	0.91797 (12)	0.0215 (3)	
H14A	0.8042	0.3624	0.8622	0.026*	
H14B	0.8174	0.3022	0.9881	0.026*	
C15	0.72582 (6)	0.31822 (13)	0.95322 (11)	0.0205 (3)	
H15A	0.7134	0.2535	1.0103	0.025*	
H15B	0.7217	0.4015	0.9908	0.025*	
C16	0.69605 (6)	0.18874 (13)	0.78029 (12)	0.0220 (3)	
H16A	0.6727	0.1888	0.7068	0.026*	
H16B	0.6820	0.1179	0.8290	0.026*	
C17	0.76314 (6)	0.17046 (14)	0.75159 (12)	0.0226 (3)	
H17A	0.7689	0.0901	0.7096	0.027*	
H17B	0.7771	0.2394	0.7003	0.027*	
C18	0.61896 (6)	0.32482 (14)	0.87638 (12)	0.0241 (3)	
H18A	0.5951	0.3275	0.8035	0.029*	
H18B	0.6062	0.2504	0.9217	0.029*	
C19	0.60671 (6)	0.44322 (14)	0.94778 (13)	0.0243 (3)	
H19A	0.6144	0.5229	0.9137	0.029*	
C20	0.58525 (6)	0.43782 (14)	1.05838 (13)	0.0234 (3)	
H20A	0.5786	0.3563	1.0892	0.028*	
C21	0.57077 (6)	0.54589 (14)	1.13783 (12)	0.0235 (3)	
C22	0.57635 (6)	0.67372 (15)	1.10234 (13)	0.0265 (3)	
H22A	0.5904	0.6926	1.0261	0.032*	
C23	0.56116 (7)	0.77284 (15)	1.17925 (14)	0.0307 (3)	
H23A	0.5651	0.8574	1.1543	0.037*	
C24	0.54012 (7)	0.74589 (16)	1.29343 (14)	0.0305 (3)	
H24A	0.5295	0.8123	1.3447	0.037*	
C25	0.53502 (7)	0.61982 (17)	1.33073 (13)	0.0318 (3)	
H25A	0.5213	0.6015	1.4073	0.038*	
C26	0.55045 (7)	0.52071 (15)	1.25361 (13)	0.0282 (3)	
H26A	0.5472	0.4363	1.2795	0.034*	
C100	0.73414 (7)	0.79844 (14)	0.43019 (13)	0.0269 (3)	
C101	0.72630 (7)	0.69456 (14)	0.51966 (13)	0.0253 (3)	
H10B	0.7510	0.6958	0.5871	0.030*	
C102	0.68628 (7)	0.60047 (13)	0.50916 (12)	0.0233 (3)	
H10C	0.6627	0.5971	0.4402	0.028*	
C103	0.67661 (6)	0.49876 (13)	0.60193 (12)	0.0222 (3)	
H1N	0.6971 (8)	0.3776 (18)	0.7921 (17)	0.027 (4)*	
H1O1	0.7076 (11)	0.851 (2)	0.279 (2)	0.054 (7)*	

### Atomic displacement parameters (Å^2^)

**Table:**

	*U*^11^	*U*^22^	*U*^33^	*U*^12^	*U*^13^	*U*^23^
O1	0.0442 (6)	0.0275 (5)	0.0254 (5)	−0.0047 (5)	−0.0059 (4)	0.0074 (4)
O2	0.0537 (8)	0.0318 (6)	0.0484 (7)	−0.0153 (6)	−0.0188 (6)	0.0135 (5)
O3	0.0385 (6)	0.0226 (5)	0.0282 (5)	−0.0041 (4)	−0.0018 (4)	−0.0003 (4)
O4	0.0407 (6)	0.0207 (5)	0.0201 (5)	−0.0006 (4)	−0.0027 (4)	0.0004 (4)
N1	0.0217 (5)	0.0202 (5)	0.0204 (5)	0.0032 (4)	−0.0016 (4)	−0.0015 (4)
N2	0.0219 (5)	0.0201 (5)	0.0180 (5)	0.0036 (4)	−0.0001 (4)	0.0003 (4)
C1	0.0185 (6)	0.0249 (7)	0.0278 (7)	0.0044 (5)	−0.0032 (5)	−0.0019 (5)
C2	0.0270 (7)	0.0267 (7)	0.0325 (8)	0.0022 (6)	0.0005 (5)	−0.0027 (6)
C3	0.0296 (8)	0.0264 (8)	0.0461 (9)	0.0017 (6)	−0.0042 (6)	−0.0054 (7)
C4	0.0295 (8)	0.0349 (8)	0.0443 (9)	0.0068 (7)	−0.0036 (6)	−0.0173 (7)
C5	0.0304 (8)	0.0484 (10)	0.0313 (8)	0.0058 (7)	0.0020 (6)	−0.0109 (7)
C6	0.0260 (7)	0.0322 (8)	0.0282 (7)	0.0035 (6)	−0.0005 (5)	−0.0012 (6)
C7	0.0219 (6)	0.0212 (6)	0.0239 (6)	0.0008 (5)	−0.0003 (5)	0.0009 (5)
C8	0.0247 (7)	0.0198 (6)	0.0280 (7)	0.0055 (5)	−0.0029 (5)	−0.0042 (5)
C9	0.0253 (7)	0.0306 (8)	0.0387 (8)	0.0035 (6)	−0.0022 (6)	−0.0071 (6)
C10	0.0272 (8)	0.0444 (10)	0.0473 (10)	0.0080 (7)	−0.0112 (7)	−0.0136 (8)
C11	0.0448 (9)	0.0386 (9)	0.0372 (9)	0.0195 (8)	−0.0199 (7)	−0.0115 (7)
C12	0.0511 (10)	0.0252 (7)	0.0282 (7)	0.0105 (7)	−0.0045 (7)	−0.0010 (6)
C13	0.0320 (7)	0.0218 (7)	0.0303 (7)	0.0024 (6)	−0.0037 (6)	−0.0006 (6)
C14	0.0234 (6)	0.0208 (6)	0.0202 (6)	0.0014 (5)	−0.0012 (5)	−0.0009 (5)
C15	0.0235 (6)	0.0216 (6)	0.0163 (6)	0.0026 (5)	−0.0015 (4)	−0.0003 (5)
C16	0.0241 (7)	0.0223 (6)	0.0197 (6)	0.0016 (5)	−0.0025 (5)	−0.0015 (5)
C17	0.0246 (7)	0.0238 (7)	0.0193 (6)	0.0043 (5)	−0.0015 (5)	−0.0023 (5)
C18	0.0205 (6)	0.0282 (7)	0.0237 (6)	0.0026 (5)	−0.0009 (5)	0.0009 (5)
C19	0.0215 (6)	0.0256 (7)	0.0260 (7)	0.0043 (5)	0.0001 (5)	0.0028 (6)
C20	0.0192 (6)	0.0246 (7)	0.0265 (7)	0.0012 (5)	−0.0012 (5)	0.0011 (5)
C21	0.0177 (6)	0.0283 (7)	0.0246 (7)	0.0001 (5)	−0.0004 (5)	−0.0003 (5)
C22	0.0239 (7)	0.0303 (8)	0.0253 (7)	−0.0010 (6)	0.0029 (5)	0.0003 (6)
C23	0.0286 (7)	0.0274 (8)	0.0360 (8)	−0.0003 (6)	0.0004 (6)	−0.0020 (6)
C24	0.0248 (7)	0.0342 (8)	0.0326 (8)	0.0021 (6)	0.0000 (5)	−0.0100 (6)
C25	0.0320 (8)	0.0402 (9)	0.0233 (7)	−0.0004 (7)	0.0034 (5)	−0.0021 (6)
C26	0.0282 (7)	0.0292 (7)	0.0271 (7)	−0.0016 (6)	0.0016 (5)	0.0012 (6)
C100	0.0291 (7)	0.0233 (7)	0.0284 (7)	0.0019 (6)	−0.0019 (5)	0.0035 (6)
C101	0.0285 (7)	0.0244 (7)	0.0230 (6)	0.0033 (6)	−0.0025 (5)	0.0028 (5)
C102	0.0297 (7)	0.0209 (6)	0.0194 (6)	0.0045 (5)	−0.0009 (5)	−0.0011 (5)
C103	0.0286 (7)	0.0183 (6)	0.0196 (6)	0.0051 (5)	0.0024 (5)	−0.0012 (5)

### Geometric parameters (Å, º)

**Table:**

O1—C100	1.3240 (19)	C12—H12A	0.9300
O1—H1O1	0.94 (3)	C13—H13A	0.9300
O2—C100	1.205 (2)	C14—C15	1.5135 (18)
O3—C103	1.2363 (18)	C14—H14A	0.9700
O4—C103	1.2837 (18)	C14—H14B	0.9700
N1—C17	1.4675 (17)	C15—H15A	0.9700
N1—C14	1.4688 (17)	C15—H15B	0.9700
N1—C7	1.4790 (17)	C16—C17	1.5194 (18)
N2—C16	1.4929 (17)	C16—H16A	0.9700
N2—C15	1.4976 (16)	C16—H16B	0.9700
N2—C18	1.5129 (17)	C17—H17A	0.9700
N2—H1N	0.943 (19)	C17—H17B	0.9700
C1—C6	1.389 (2)	C18—C19	1.497 (2)
C1—C2	1.395 (2)	C18—H18A	0.9700
C1—C7	1.5238 (19)	C18—H18B	0.9700
C2—C3	1.385 (2)	C19—C20	1.327 (2)
C2—H2A	0.9300	C19—H19A	0.9300
C3—C4	1.389 (3)	C20—C21	1.472 (2)
C3—H3A	0.9300	C20—H20A	0.9300
C4—C5	1.382 (3)	C21—C26	1.397 (2)
C4—H4A	0.9300	C21—C22	1.398 (2)
C5—C6	1.398 (2)	C22—C23	1.387 (2)
C5—H5A	0.9300	C22—H22A	0.9300
C6—H6A	0.9300	C23—C24	1.389 (2)
C7—C8	1.5267 (19)	C23—H23A	0.9300
C7—H7A	0.9800	C24—C25	1.385 (2)
C8—C13	1.393 (2)	C24—H24A	0.9300
C8—C9	1.394 (2)	C25—C26	1.390 (2)
C9—C10	1.389 (2)	C25—H25A	0.9300
C9—H9A	0.9300	C26—H26A	0.9300
C10—C11	1.380 (3)	C100—C101	1.487 (2)
C10—H10A	0.9300	C101—C102	1.323 (2)
C11—C12	1.391 (3)	C101—H10B	0.9300
C11—H11A	0.9300	C102—C103	1.5010 (19)
C12—C13	1.392 (2)	C102—H10C	0.9300
			
C100—O1—H1O1	110.6 (15)	N2—C15—H15A	109.7
C17—N1—C14	107.31 (10)	C14—C15—H15A	109.7
C17—N1—C7	112.44 (10)	N2—C15—H15B	109.7
C14—N1—C7	109.95 (11)	C14—C15—H15B	109.7
C16—N2—C15	110.08 (10)	H15A—C15—H15B	108.2
C16—N2—C18	109.73 (11)	N2—C16—C17	111.02 (11)
C15—N2—C18	112.20 (10)	N2—C16—H16A	109.4
C16—N2—H1N	106.2 (11)	C17—C16—H16A	109.4
C15—N2—H1N	108.8 (12)	N2—C16—H16B	109.4
C18—N2—H1N	109.7 (11)	C17—C16—H16B	109.4
C6—C1—C2	119.00 (14)	H16A—C16—H16B	108.0
C6—C1—C7	119.86 (13)	N1—C17—C16	109.63 (11)
C2—C1—C7	121.14 (13)	N1—C17—H17A	109.7
C3—C2—C1	120.71 (14)	C16—C17—H17A	109.7
C3—C2—H2A	119.6	N1—C17—H17B	109.7
C1—C2—H2A	119.6	C16—C17—H17B	109.7
C2—C3—C4	120.00 (16)	H17A—C17—H17B	108.2
C2—C3—H3A	120.0	C19—C18—N2	111.86 (11)
C4—C3—H3A	120.0	C19—C18—H18A	109.2
C5—C4—C3	119.90 (15)	N2—C18—H18A	109.2
C5—C4—H4A	120.1	C19—C18—H18B	109.2
C3—C4—H4A	120.1	N2—C18—H18B	109.2
C4—C5—C6	120.11 (15)	H18A—C18—H18B	107.9
C4—C5—H5A	119.9	C20—C19—C18	121.87 (13)
C6—C5—H5A	119.9	C20—C19—H19A	119.1
C1—C6—C5	120.28 (15)	C18—C19—H19A	119.1
C1—C6—H6A	119.9	C19—C20—C21	127.53 (14)
C5—C6—H6A	119.9	C19—C20—H20A	116.2
N1—C7—C1	111.23 (11)	C21—C20—H20A	116.2
N1—C7—C8	110.08 (11)	C26—C21—C22	118.15 (14)
C1—C7—C8	110.24 (11)	C26—C21—C20	119.13 (13)
N1—C7—H7A	108.4	C22—C21—C20	122.72 (13)
C1—C7—H7A	108.4	C23—C22—C21	120.93 (13)
C8—C7—H7A	108.4	C23—C22—H22A	119.5
C13—C8—C9	119.22 (14)	C21—C22—H22A	119.5
C13—C8—C7	121.79 (13)	C22—C23—C24	120.09 (15)
C9—C8—C7	118.95 (13)	C22—C23—H23A	120.0
C10—C9—C8	120.44 (16)	C24—C23—H23A	120.0
C10—C9—H9A	119.8	C25—C24—C23	119.81 (14)
C8—C9—H9A	119.8	C25—C24—H24A	120.1
C11—C10—C9	120.23 (16)	C23—C24—H24A	120.1
C11—C10—H10A	119.9	C24—C25—C26	119.97 (14)
C9—C10—H10A	119.9	C24—C25—H25A	120.0
C10—C11—C12	119.84 (15)	C26—C25—H25A	120.0
C10—C11—H11A	120.1	C25—C26—C21	121.03 (14)
C12—C11—H11A	120.1	C25—C26—H26A	119.5
C11—C12—C13	120.23 (16)	C21—C26—H26A	119.5
C11—C12—H12A	119.9	O2—C100—O1	123.66 (14)
C13—C12—H12A	119.9	O2—C100—C101	122.27 (14)
C12—C13—C8	120.03 (15)	O1—C100—C101	114.07 (13)
C12—C13—H13A	120.0	C102—C101—C100	123.96 (14)
C8—C13—H13A	120.0	C102—C101—H10B	118.0
N1—C14—C15	110.31 (11)	C100—C101—H10B	118.0
N1—C14—H14A	109.6	C101—C102—C103	123.90 (13)
C15—C14—H14A	109.6	C101—C102—H10C	118.0
N1—C14—H14B	109.6	C103—C102—H10C	118.0
C15—C14—H14B	109.6	O3—C103—O4	124.57 (13)
H14A—C14—H14B	108.1	O3—C103—C102	118.50 (13)
N2—C15—C14	110.00 (10)	O4—C103—C102	116.93 (12)
			
C6—C1—C2—C3	0.1 (2)	C7—N1—C14—C15	−173.18 (10)
C7—C1—C2—C3	−179.34 (13)	C16—N2—C15—C14	54.01 (14)
C1—C2—C3—C4	0.1 (2)	C18—N2—C15—C14	176.51 (11)
C2—C3—C4—C5	−0.4 (2)	N1—C14—C15—N2	−60.16 (14)
C3—C4—C5—C6	0.6 (2)	C15—N2—C16—C17	−54.01 (14)
C2—C1—C6—C5	0.1 (2)	C18—N2—C16—C17	−177.96 (10)
C7—C1—C6—C5	179.51 (13)	C14—N1—C17—C16	−63.17 (14)
C4—C5—C6—C1	−0.4 (2)	C7—N1—C17—C16	175.81 (11)
C17—N1—C7—C1	−50.90 (15)	N2—C16—C17—N1	59.32 (14)
C14—N1—C7—C1	−170.41 (11)	C16—N2—C18—C19	176.75 (11)
C17—N1—C7—C8	−173.39 (11)	C15—N2—C18—C19	54.05 (15)
C14—N1—C7—C8	67.10 (14)	N2—C18—C19—C20	−117.16 (14)
C6—C1—C7—N1	119.21 (14)	C18—C19—C20—C21	−179.45 (13)
C2—C1—C7—N1	−61.36 (17)	C19—C20—C21—C26	−177.76 (14)
C6—C1—C7—C8	−118.38 (14)	C19—C20—C21—C22	2.4 (2)
C2—C1—C7—C8	61.04 (17)	C26—C21—C22—C23	−1.1 (2)
N1—C7—C8—C13	36.25 (18)	C20—C21—C22—C23	178.80 (14)
C1—C7—C8—C13	−86.82 (16)	C21—C22—C23—C24	0.1 (2)
N1—C7—C8—C9	−145.97 (13)	C22—C23—C24—C25	0.7 (2)
C1—C7—C8—C9	90.96 (16)	C23—C24—C25—C26	−0.5 (2)
C13—C8—C9—C10	1.0 (2)	C24—C25—C26—C21	−0.5 (2)
C7—C8—C9—C10	−176.85 (14)	C22—C21—C26—C25	1.3 (2)
C8—C9—C10—C11	−0.1 (3)	C20—C21—C26—C25	−178.60 (13)
C9—C10—C11—C12	−0.6 (3)	O2—C100—C101—C102	173.65 (16)
C10—C11—C12—C13	0.4 (2)	O1—C100—C101—C102	−5.8 (2)
C11—C12—C13—C8	0.5 (2)	C100—C101—C102—C103	−177.35 (13)
C9—C8—C13—C12	−1.2 (2)	C101—C102—C103—O3	−175.41 (14)
C7—C8—C13—C12	176.58 (13)	C101—C102—C103—O4	4.2 (2)
C17—N1—C14—C15	64.23 (13)		

### Hydrogen-bond geometry (Å, º)

**Table:**

*D*—H···*A*	*D*—H	H···*A*	*D*···*A*	*D*—H···*A*
N2—H1*N*···O4	0.943 (19)	1.740 (19)	2.6750 (15)	170.9 (17)
O1—H1*O*1···O4^i^	0.94 (3)	1.70 (3)	2.6270 (15)	168 (2)
C18—H18*B*···O3^ii^	0.97	2.56	3.3839 (18)	143
C15—H15*A*···O3^ii^	0.97	2.47	3.3463 (18)	151
C15—H15*B*···O2^iii^	0.97	2.46	3.1941 (19)	132

Symmetry codes: (i) *x*, −*y*+3/2, *z*−1/2; (ii) *x*, −*y*+1/2, *z*+1/2; (iii) *x*, −*y*+3/2, *z*+1/2.
